# Trends in Engagement With Opioid Use Disorder Treatment Among Medicaid Beneficiaries During the COVID-19 Pandemic

**DOI:** 10.1001/jamahealthforum.2022.0093

**Published:** 2022-03-11

**Authors:** Alyssa Shell Tilhou, Laura Dague, Brendan Saloner, Daniel Beemon, Marguerite Burns

**Affiliations:** 1 Department of Family Medicine, Boston University School of Medicine/Boston Medical Center, Boston, Massachusetts; 2 Department of Public Service and Administration, Texas A&M University, College Station, Texas; 3 Department of Health Policy and Management, Johns Hopkins University, Baltimore, Maryland; 4 Department of Economics, University of Wisconsin-Madison, Madison; 5 Department of Population Health Sciences, University of Wisconsin-Madison, Madison

## Abstract

**Importance:**

Disruptions in care during the COVID-19 pandemic may have decreased access to care for patients with opioid use disorder.

**Objective:**

To examine trends in opioid use disorder treatment including buprenorphine possession, urine drug testing, and opioid treatment program services during the COVID-19 public health emergency.

**Design, Setting, and Participants:**

This cohort study included 6453 parent and childless adult Medicaid beneficiaries, aged 18 to 64 years, with opioid use disorder and continuous enrollment from December 1, 2018, to September 30, 2020, in Wisconsin. Logistic regression compared differences in study outcomes before, early, and later in the COVID-19 public health emergency. Analyses were conducted from January 2021 to October 2021.

**Exposures:**

Early (March 16, 2020, to May 15, 2020) and later (May 16, 2020, to September 30, 2020) in the public health emergency.

**Main Outcomes and Measures:**

Person-week outcomes included possession of buprenorphine, completion of outpatient urine drug testing, and receipt of opioid treatment program services.

**Results:**

The final cohort of 6453 participants included 3986 (61.8%) childless adults; 5741 (89%) were younger than 50 years, 3435 (53.2%) were women, 5036 (78.0%) White, and 22.0% were racial and ethnic minority groups (American Indian, 269 [4.2%]; Asian, 26 [0.4%]; Black, 458 [7.1%]; Hispanic, 292 [4.5%]; Pacific Islander, 1 [.02%]; Multiracial, 238 [3.7%]). Overall, 2858 (44.3%), 5074 (78.6%), and 2928 (45.4%) received buprenorphine, urine drug testing, or opioid treatment program services during the study period, respectively. Probability of buprenorphine possession did not change in the early or later part of the public health emergency. Probability of urine drug testing initially decreased (marginal effect [ME], –0.04; 95% CI, –0.04 to –0.03; *P* < .001) and then partially recovered in the later public health emergency (ME, –0.02; 95% CI, –0.03 to –0.02; *P* < .001). Probability of opioid treatment program services followed a similar pattern, with an early decrease (ME, –0.05; 95% CI, –0.05 to –0.04; *P* < .001) followed by partial recovery (ME, –0.02; 95% CI, –0.03 to –0.02; *P* < .001).

**Conclusions and Relevance:**

In a sample of continuously enrolled adult Medicaid beneficiaries, the COVID-19 public health emergency was not associated with decreased probability of buprenorphine possession, but was associated with decreased probability of urine drug testing and opioid treatment program services. These findings suggest patients in office-based settings retained access to buprenorphine despite decreased on-site services like urine drug tests, whereas patients at opioid treatment programs experienced greater disruption in care. Given the importance of medications for opioid use disorder in preventing overdose, policy makers should consider permanent policy changes based on lessons learned from the public health emergency to enable ongoing enhanced access to these medications.

## Key Points

**Question:**

During the COVID-19 public health emergency, did patients with opioid use disorder experience decreased access to opioid use disorder treatment?

**Findings:**

In this cohort study of 6453 Medicaid beneficiaries with opioid use disorder in Wisconsin, buprenorphine possession remained stable at the onset and for the first 6 months of the public health emergency. In contrast, completion of urine drug tests and receipt of opioid treatment program services declined with the onset of the public health emergency and recovered partially 6 months into the public health emergency.

**Meaning:**

The findings of this study suggest that the COVID-19 public health emergency did not disrupt access to buprenorphine but did disrupt urine drug testing and access to opioid treatment program services.

## Teaser

This cohort study examines whether Medicare patients in Wisconsin with opioid use disorder experienced decreased access to opioid use disorder treatment during the COVID-19 pandemic.

## Introduction

Opioid use disorder (OUD) is a chronic relapsing disease that continues to take lives at an escalating rate.^1,2^ Against the backdrop of the COVID-19 public health emergency (PHE), opioid-involved overdose deaths reached a record high of 69 710 in 2020.^3^ Opioid agonist therapy with buprenorphine and methadone are central to the treatment of OUD. Opioid agonist therapy is associated with improved outcomes such as treatment retention,^4^ reduced use,^4^ and all-cause and opioid overdose mortality.^5^ As a result, access to opioid agonist therapy represents a critical intervention in addressing the opioid epidemic.

The COVID-19 pandemic in early 2020 created barriers to in-person care, potentially disrupting access to opioid agonist therapy. Access to these medications occurs through 2 main settings: office-based opioid treatment, where patients can receive buprenorphine prescriptions, and federally regulated opioid treatment programs (OTPs), where patients can receive dispensed methadone or buprenorphine, usually in the form of daily dosing; more rarely in the form of take-home doses—OTP-dispensed doses for future use. At the start of the pandemic, many clinics swiftly reduced provision of in-person care.^6^ In tandem, many patients avoided or delayed in-person care.^7,8^ In combination, these factors may have reduced access to opioid agonist therapy. Thus far, analyses suggest that most patients receiving buprenorphine were able to retain access to medication,^9,10,11^ though not all studies agree.^12^ In contrast, access to methadone likely declined.^13^ Notably, little research has focused on access to treatment among Medicaid patients,^11^ a population particularly vulnerable to the challenges posed by the pandemic owing to higher rates of financial, housing, and employment insecurity.^14,15^

Through reductions in in-person care, the pandemic may have reduced access to other core addiction services like urine drug testing (UDT). Although incorporation of mobile unit UDT occurred in some settings,^16^ reports suggest that ambulatory UDT decreased during the pandemic.^10,17^ A comprehensive assessment of UDT trends during the PHE has not yet been performed.

This study examined the association of the COVID-19 PHE with access to treatment for Wisconsin Medicaid patients with established OUD seeking care in both office-based and OTP settings. To examine access to office-based opioid treatment, we evaluated the effect of the PHE on buprenorphine possession and UDT. Alongside these outcomes, we examined trends in OTP service receipt. Together, these analyses present a more holistic view of patients’ access to OUD care during the COVID-19 PHE.

## Methods

### Data Source

Study data sources included Wisconsin Medicaid administrative, enrollment and claims data from December 2018 to September 2020. We used claims to identify beneficiaries with an OUD diagnosis for cohort construction. Demographic characteristics of the cohort, obtained from enrollment data at baseline in December 2018, included Medicaid eligibility category, age, sex, race, ethnicity, education, and geography. In general, race and ethnicity are obtained via self-identification but occasionally reported by caseworkers.

### Study Population

The study cohort included nonpregnant, nondisabled adults ages 18 to 64 years who were eligible for Wisconsin Medicaid through 1 of 2 pathways: parent/caretaker or adult without dependent children (henceforth, “childless adult”), and who were continuously enrolled from December 2018 to September 2020, 15 months before and roughly 6 months after declaration of the PHE. The final cohort of 6453 participants included 3986 (61.8%) childless adults; 5741 (89%) were younger than 50 years, 3435 (53.2%) were women, 5036 (78.0%) were White, and 22.0% were in racial and ethnic minority groups (American Indian, 269 [4.2%]; Asian, 26 [0.4%]; Black, 458 [7.1%]; Hispanic, 292 [4.5%]; Pacific Islander, 1 [.02%]; Multiracial, 238 [3.7%]). Individuals who turned age 65 years during the study period were excluded. We required continuous enrollment to focus on the association of the PHE with access to care among those with access to treatment prior to the PHE, rather than among new enrollees or treatment naive individuals. Continuous enrollment was defined as an enrollment gap of less than 1 month. For cohort inclusion, we required at least 1 claim with an OUD diagnosis in the 6-months prior to the study period (June 2018 to November 2018) in claims from outpatient, inpatient, or emergency department services. By requiring diagnosis prior to the study period, we constructed a sample with established OUD. Continuous enrollment in this preperiod was not required. We identified OUD diagnosis using the *International Statistical Classification of Diseases and Related Health Problems, Tenth Revision (ICD-10)* codes F11.xxx. See eTables 1 to 3 in the Supplement for additional detail regarding the underlying population and cohort construction.

### Outcome Assessment and Variables

Outpatient buprenorphine posession was defined as receipt of any prescribed buprenorphine in the week. We constructed our measure of outpatient buprenorphine posession using pharmacy claims data and a definition adapted from the Medicaid Outcomes Distributed Research Network.^18^ In this measure, we excluded buprenorphine patches as well as formulations billed under a Healthcare Common Procedure Coding System (HCPCS) code to minimize capture of buprenorphine dispensed at an OTP. In addition, we used medical claims to construct 2 additional outcome measures: receipt of OTP services in the week and completion of an outpatient (non-OTP) urine drug screen or test (UDT) in the week. Receipt of OTP services was defined by any claim associated with an OTP billing entity. In this study, OTP-dispensed buprenorphine claims represented a near-zero proportion of OTP claims across study periods and are included in the OTP services measure. See eAppendix 1 in the Supplement for additional information regarding measure specifications.

### Statistical Analysis

Baseline characteristics are summarized for the sample. Rates of buprenorphine possession, UDT completion, and OTP services receipt were estimated as proportion of the cohort at the person-week level. To construct the person-week measure, we divided each calendar month into quarters of 7 or 8 days to align health care data with the calendar month. This calendar reconstruction is required because Medicaid enrollment is by calendar month, prohibiting the use of a standard 7-day week to investigate health care utilization. This measure is described further in eAppendix 2 in the Supplement.

Using the person-week as the unit of analysis, we estimated the rate of buprenorphine possession, UDT completion, and OTP services receipt across 3 study periods defined by the following dates: pre-PHE (December 1, 2018-March 15, 2020), early PHE (March 16, 2020-May 15, 2020), and later PHE (May 16, 2020-September 30, 2020). March 16, 2020, marks the first day of the first person-week after the Governor of Wisconsin declared a public health emergency in response to the COVID-19 pandemic.^19^ The other dates are based on patterns evident in the data (Figure). We conducted logistic regression to test for differences in the probability of each outcome in the early and later PHE relative to pre-PHE, clustering the standard error at the individual level. We present results as marginal effects. Sensitivity analyses were performed adjusting for seasonality, select outlier points, and scaling by number of weekdays per person-week. Analyses were conducted using Stata statistical software (version 17.0; Stata Corp, LLP). The statistical significance level was set at 0.05. Analyses were conducted from January 2021 to October 2021.

**Figure.**
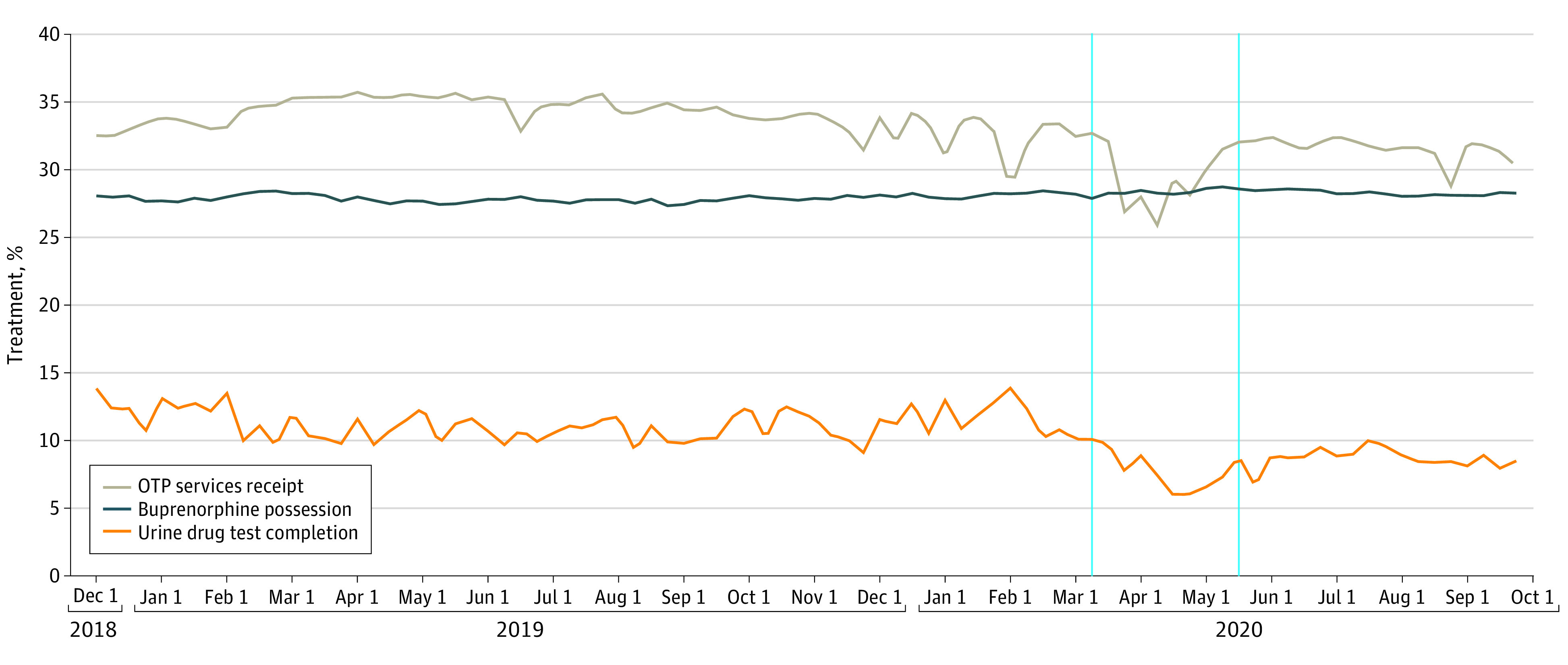
Trends in OUD Treatment Before and During the COVID-19 PHE The vertical blue lines indicate the start of the early PHE period (March 16, 2020) and the later PHE period (April 16, 2020). OTP Indicates opioid treatment program; OUD, opioid use disorder; PHE, public health emergency.

This study was determined exempt from review and informed consent by the University of Wisconsin’s institutional review board (common rule, category 5). This study followed the Strengthening the Reporting of Observational Studies in Epidemiology (STROBE) reporting guidelines.

## Results

We identified 115 638 individuals who met the age, Medicaid eligibility pathway and enrollment continuity criteria. Our analytic sample of 6453 individuals represents the subset of this cohort who received an OUD diagnosis during the 6 months prior to the study. Results characterizing the analytic sample are presented in Table 1. Most individuals received Medicaid via the childless adult pathway (3986 [61.8%]). Most were women (3435 [53.2%]) and White (5036 [78.0%]). Racial and ethnic minority groups included 269 (4.2%) American Indian, 26 (0.4%) Asian, 458 (7.1%) Black, 292 (4.5%) Hispanic, 1 (0.02%) Pacific Islander, and 238 (3.7%) multiracial, with 133 (2.1%) missing race and ethnicity information. Only 712 (11.0%) were aged 50 years or older. Most had graduated high school (4000 [62.0%]), lived in urban areas (4534 [70.3%]), and earned less than 50% of the federal poverty line (5520 [85.5%]). Overall, 2858 (44.3%) claimed a prescription for buprenorphine, 5074 (78.6%) completed UDT, and 2928 (45.4%) received OTP services during the study period. Overall, 79.1% either filled a buprenorphine prescription or received OTP services during the study period.

**Table 1.** Characteristics of 6453 Adult Wisconsin Medicaid Patients With Opioid Use Disorder Enrolled December 1, 2018, to September 30, 2020^a^

Characteristic	No. (%)
Eligibility category	
Childless adults	3986 (61.8)
Parents	2467 (38.2)
Treatment indicators	
Buprenorphine patient	2858 (44.3)
UDT patient	5074 (78.6)
OTP patient	2928 (45.4)
Age group, y	
18-34	2832 (43.9)
35-49	2909 (45.1)
≥50	712 (11.0)
Race and ethnicity	
American Indian	269 (4.2)
Asian	26 (0.4)
Black	458 (7.1)
Hispanic	292 (4.5)
Pacific Islander	1 (0.02)
White	5036 (78.0)
Multiracial	238 (3.7)
Sex	
Female	3435 (53.2)
Male	3018 (46.8)
Geography	
Urban	4534 (70.3)
Rural	1368 (21.2)
Education	
<High school	1297 (20.1)
Graduated high school	4000 (62.0)
Income	
≤50 FPL	5520 (85.5)
50%-100% FPL	933(14.5)

Abbreviations: FPL, federal poverty level; OTP, opioid treatment program; UDT, urine drug test.

^a^ Nonconstant demographic variables are counted from first observed month in sample period. Missing values for race, 133 (2.1%), geography, 551 (8.5%), and education, 1156 (17.9%).

The Figure demonstrates buprenorphine possession, UDT completion, and OTP services receipt before and during the PHE. Buprenorphine possession remained stable at about 28% of the sample leading up to and during the PHE. In contrast, in the early PHE, the proportion of individuals completing UDTs decreased and only partially recovered to prepandemic levels by the later PHE. During the study period, OTP services receipt followed a similar pattern as UDTs, with a substantial decrease in the early PHE and partial recovery in the later PHE.

Table 2 presents descriptive statistics about the weekly rate of possessing buprenorphine, completing a UDT and receiving OTP services prior to and during the PHE. Prior to the PHE, the mean rate of buprenorphine possession was 0.28 (range, 0.27-0.29; standard error [SE], <0.01) and remained essentially unchanged in the early and later PHE. In contrast, the mean rate of UDT completion decreased from 0.11 prior to the PHE (range, 0.09-0.14; SE, <0.01) to 0.07 (range, 0.06-0.10; SE, <0.01) and 0.09 (range, 0.07-0.10; SE, <0.01) in the early and later PHE, respectively. The mean rate of OTP services receipt prior to the PHE decreased from 0.34 (range, 0.29-0.36; SE, <0.01) to 0.29 (range, 0.26-0.32; SE, <0.01) and 0.32 (range, 0.29-0.33; SE, <0.01) during the early and later PHE, respectively.

**Table 2.** Rates of Treatment Receipt Before and During the COVID-19 Public Health Emergency^a^

Time period^b^	Buprenorphine possession	UDT completion	OTP services receipt
	Mean (range) [SE]	Mean (range) [SE]	Mean (range) [SE]
Pre-PHE	0.28 (0.27-0.29) [<0.01]	0.11 (0.09-0.14) [<0.01]	0.34 (0.29-0.36) [<0.01]
Early PHE	0.29 (0.28-0.29) [<0.01]	0.07 (0.06-0.10) [<0.01]	0.29 (0.26-0.32) [0.01]
Later PHE	0.28 (0.28-0.29) [<0.01]	0.09 (0.07-0.10) [<0.01]	0.32 (0.29-0.33) [<0.01]

Abbreviations: OTP, opioid treatment program; PHE, public health emergency; SE, standard error; UDT, urine drug test.

^a^ Rates represent the proportion of the cohort receiving treatment in the week.

^b^ Pre-PHE includes December 1, 2018, to March 15, 2020; early PHE, March 16, 2020, to May 15, 2020; and later PHE, May 16, 2020 to September 30, 2020.

As shown in the Figure, March 16, 2020, and May 16, 2020, were selected as cutoff points to define the study periods. Table 3 presents results from multivariable logistic regression. There was no significant change in the probability of buprenorphine possession in the early or later PHE compared with pre-PHE (early PHE marginal effect [ME], 0.005; 95% CI, –0.000 to 0.010; *P* = .07; later PHE ME, 0.004; 95% CI, –0.002 – 0.010; *P* = .17). In contrast, the early PHE was associated with a significant decrease in receiving UDT and OTP services. Specifically, the probability of UDT in the early and later PHE was 3.6 percentage points (pp) (95% CI, –0.039 to –0.033; *P* < .001) and 2.4 pp (95% CI, –0.028 to –0.021; *P* < .001) lower than pre-PHE, respectively. The probability of receiving OTP services in the early and later PHE was 5.0 pp (95% CI, –0.055 to –0.044; *P* < .001) and 2.4 pp (95% CI, –0.029 to –0.018; *P* < .001) lower than pre-PHE, respectively.

**Table 3.** Marginal Effect on the Probability of Receiving Treatment Before and During the COVID-19 Public Health Emergency

Time period indicators	Buprenorphine possession		UDT completion		OTP services receipt	
	ME (95% CI)	*P* value	ME (95% CI)	*P* value	ME (95% CI)	*P* value
Pre-PHE: 12/1/18 - 3/15/20 (Reference)	0.28 (0.27 to 0.29)	<.001	0.11 (0.11 to 0.12)	<.001	0.34 (0.33 to 0.35)	<.001
Early PHE: 3/16/20 - 5/15/20	0.01 (–0.00 to 0.01)	.07	–0.04 (–0.04 to –0.03)	<.001	–0.05 (–0.05 to –0.04)	<.001
Later PHE: 5/16/20 - 9/30/20	0.004 (–0.002 to 0.01)	.17	–0.02 (–0.03 to –0.02)	<.001	–0.02 (–0.03 to –0.02)	<.001

Abbreviations: ME, marginal effect; OTP, opioid treatment program; PHE, public health emergency; UDT, urine drug test.

Sensitivity analyses were performed to assess the effect of an outlier week for the UDT and OTP rates (Figure). In week 57, the rate of UDT completion was 0.14 compared with a pre-PHE mean of 0.11, whereas the rate of OTP services receipt was 0.29 compared with a pre-PHE mean of 0.34. To assess the effect of this outlier week, we repeated regression analyses for the UDT and OTP outcomes with this week excluded. Coefficients and *P* values were essentially unchanged. Additional sensitivity analyses controlling for seasonality across all 3 outcomes and number of weekdays in the person-week for both UDT and OTP outcomes did not affect regression results.

## Discussion

In a sample of Wisconsin Medicaid beneficiaries with established OUD, we found that possession of buprenorphine remained stable in the early and later periods of the COVID-19 PHE, whereas rates of UDT and OTP services receipt decreased in the early PHE and partially recovered in the later PHE. Our findings suggest that patients with OUD were able to maintain access to buprenorphine during the pandemic through office-based settings despite disruptions in on-site care as evidenced by reduced UDT. These findings on buprenorphine stability for Medicaid patients during the PHE are in line with results from other samples.^9,10,11^ In contrast with buprenorphine possession stability, we found that receipt of OTP services decreased in the early PHE, and only partially recovered in the later PHE. Specifically, the average rate of patients receiving OTP services in the week dropped by over 14% from 0.34 prior to the PHE to 0.29 early in the PHE. These findings suggest that OTP patients may have experienced considerable disruption in access to care.

Interpreting these diverging trends in access to care through office-based and OTP settings requires attention to the regulatory context of OUD treatment in the early pandemic. Prior to the pandemic, federal regulations required an in-person examination to initiate receipt of controlled substances. After the PHE declaration, federal officials temporarily modified these regulations to allow initial and ongoing prescribing of controlled substances, including buprenorphine, via telemedicine or telephone visit.^20^ New patients seeking methadone at OTPs, however, were not exempt from an in-person evaluation.^20,21,22^

In light of these regulatory changes, maintained rates of buprenorphine possession may represent rapid expansion of telehealth services to enhance access for patients seeking care in office-based settings.^23,24^ Relative to OTPs, office-based settings may have been particularly well-positioned to expand telehealth services. Office-based opioid treatment often takes place in physician practices that care for a wider spectrum of disease entities than OTPs, which are focused exclusively on OUD.^25^ As a result, addiction care in office-based settings may have benefited from transdisciplinary efforts to incorporate telehealth across service sectors.^26,27^

Regulatory changes may have had a more muted effect in OTP settings for several reasons. Although some OTPs did incorporate telehealth services,^23,28,29^ new patients seeking methadone treatment were still required to complete in-person evaluations.^20^ Since methadone for OUD is only available through an OTP, the required in-person examination may have deterred patients from seeking care thereby contributing to decreased receipt of OTP services during the PHE. In addition, although buprenorphine prescriptions through office-based settings are sent to community pharmacies as a multiday supply, methadone is usually prescribed and dispensed on-site as a daily administered dose. This logistical difference in medication access, compounded by fear of contagion, likely created a context in which telehealth eased access to buprenorphine in a way that could not be replicated for methadone. These factors may explain why rates of buprenorphine possession remained stable while receipt of OTP services fell. Decreased outpatient UDT completion during the PHE provides important circumstantial evidence that patients were likely receiving office-based opioid treatment via telehealth instead of presenting for in-person care. Significantly, the allowance to initiate controlled substances via telehealth is based on a temporary exception to the Controlled Substances Act afforded by the PHE.^21,30^ Reversal of this allowance after the PHE could have considerable consequences for patients’ access to opioid agonist therapy.

It is important to note that observed decreases in receipt of OTP services could, in part, reflect an increase in take-home doses rather than a reduction in care. At the onset of the PHE, federal policy makers eased restrictions on the provision of take-home doses.^20,31^ Specifically, states were given permission to request up to 14 and 28 days of take-home medication for less stable and stable patients, respectively,^32^ according to specific eligibility criteria.^33^ Uptake of these loosened restrictions may have created opportunity for patients in OTPs to continue receiving care while presenting less frequently. Preliminary evidence suggests high variability in implementation of loosened take-home restrictions, with some clinics not reporting adoption and other clinics reporting substantial increases in take-home doses dispensed.^28,34,35^ As a result, our trends may underestimate actual receipt of OTP services during the PHE. Of note, expansion of OTP telehealth services may have contributed to reduced levels of on-site care, but would not explain the observed decrease in engagement because our measure of OTP services includes both face-to-face and telehealth visits.

Despite the apparent stability of buprenorphine possession, it is still possible that the health system was not adequately meeting demand for office-based opioid treatment. Pandemic associated stressors may have triggered worsening individual substance use for many patients, as suggested by rising overdose rates.^3,36,37^ In this way, we might have expected increased need for OUD treatment and therefore increased rates of buprenorphine possession even in our sample of patients with established OUD. From another angle, although pharmacotherapy is important for treating OUD,^4,5^ medication possession does not confirm access to wraparound services like behavioral health, transportation vouchers, and housing assistance, which are often provided on-site by diverse team members in the context of a clinician visit. As such, observed rates of buprenorphine possession cannot speak to receipt of these important psychosocial resources.

Uniquely, this study contributes findings to the literature on trends in urine drug testing. We demonstrate that prior to the pandemic, the average weekly proportion of patients receiving UDT was 0.11. However, in the early PHE, this rate dropped by over 35% and only partially recovered to prepandemic levels by later in the PHE. These trends reflect recommendations from professional organizations like the American Society of Addiction Medicine to suspend routine UDT during the pandemic to minimize exposure risk for patients.^38^ Notably, urine drug testing has long held a central role in the standard treatment of substance use disorders.^39,40^ However, pandemic barriers to on-site services have energized some practitioners to advocate for deemphasis of UDT, citing equivocal evidence of benefits as well as potential harms.^41,42^ Whether UDT rates in Wisconsin will return to prepandemic levels is yet to be determined.

### Limitations

By requiring continuous Medicaid enrollment, we were able to more confidently report within-person changes in the probability of buprenorphine possession, UDT completion, and OTP services receipt during the PHE. However, this cohort construction excludes individuals with inconsistent enrollment, such as those with job instability or housing instability who may have responded differently to the PHE. In this way, the requirement for continuous enrollment limits the generalizability of our findings. This limitation is somewhat softened by maintenance of effort protections provided by the Families First Coronavirus Response Act, which essentially prohibited disenrollment for Wisconsin Medicaid beneficiaries during the PHE.^43^ Consequently, our sample includes beneficiaries who may otherwise have lost Medicaid coverage owing to increased income or other life changes.

This study focused on individuals with OUD diagnosed in the 6 months before December 2018. Consequently, these findings may not generalize to individuals diagnosed after November 2018 or who receive OUD care infrequently. We explored the degree to which timing of diagnosis ascertainment may have influenced cohort characteristics and found similar characteristics in a cohort captured more proximally to the PHE (eTable 3 in the Supplement).

Our analysis used claims data to identify individuals with OUD, mirroring other research on OUD in Medicaid populations.^18,44^ This approach carries known limitations, like failing to identify patients receiving treatment without diagnoses while capturing others with misuse but not addiction.^45,46^

Although we identified changes in OTP engagement associated with the PHE, we did not observe the specific mechanism(s) by which these changes occurred. For example, decreases in OTP services receipt could represent increased rates of relapse (and no OTP services), increased take-home supply, or transition from an OTP to another setting such as office-based opioid treatment. Our analyses also did not assess to what degree telehealth served to maintain buprenorphine possession in comparison with OTP engagement. Future research should attempt to understand the mechanisms explaining the observed decrease in OTP services.

## Conclusions

This cohort study found that Wisconsin Medicaid patients with previously diagnosed OUD were able to maintain comparable levels of buprenorphine possession before and during the COVID-19 PHE despite challenges to on-site care as demonstrated by decreased urine drug testing. In contrast, we found that entrance into the PHE was associated with decreased receipt of OTP services, and that this trend only partially recovered in the later PHE. These findings carry significant implications for patients living with OUD: the period following discontinuation of methadone or buprenorphine carries increased risk of all-cause and opioid overdose mortality.^5^ Further research should explore the mechanisms by which access to medications for OUD changed during the PHE, such as through telehealth services, and whether shifts to alternative care models (eg, telehealth) and/or reduced on-site services (eg, UDT) affected salient outcomes such as relapse or overdose. Furthermore, given the importance of medications for OUD in preventing overdose, policy makers should consider permanent policy changes based on lessons learned from the PHE to enable ongoing enhanced access to these life-saving medications.

## Notes

Supplement. eTable 1. Sample Construction eTable 2. Comparison of Analytic Sample to Underlying Population eTable 3. Comparison of Current Cohort to an Alternative Future Staggered Cohort eAppendix 1. Definition of Outcome Measures eAppendix 2. Definition of Person-Week Measure eReferences
