# Supplementary material for: Trends in Engagement With Opioid Use Disorder Treatment Among Medicaid Beneficiaries During the COVID-19 Pandemic
Source: JAMA Health Forum. 2022 Mar 11;3(3):e220093. doi: 10.1001/jamahealthforum.2022.0093 (PMC8917419; doi:10.1001/jamahealthforum.2022.0093)
Supplement: Supplement. — eTable 1. Sample Construction eTable 2. Comparison of Analytic Sample to Underlying Population eTable 3. Comparison of Current Cohort to an Alternative Future Staggered Cohort eAppendix 1. Definition of Outcome Measures eAppendix 2. Definition of Person-Week Measure eReferences [file jamahealthforum-e220093-s001.pdf]

Supplemental Online Content

Tilhou AS, Dague L, Saloner B, Beemon D, Burns M. Trends in engagement with opioid use disorder treatment among Medicaid beneficiaries during the COVID-19 pandemic. *JAMA Health Forum*. 2022;3(3):e220093. doi:10.1001/jamahealthforum.2022.0093

eTable 1. Sample Construction

eTable 2. Comparison of Analytic Sample to Underlying Population

eTable 3. Comparison of Current Cohort to an Alternative Future Staggered Cohort

eAppendix 1. Definition of Outcome Measures

eAppendix 2. Definition of Person-Week Measure

eReferences

This supplemental material has been provided by the authors to give readers additional information about their work.

© 2022 Tilhou AS, et al. *JAMA Health Forum.*

eAppendix1

eTable 1. Sample Construction

**Inclusion/Exclusion Criterion**	**Unique Individuals**
(1) Population: Individuals ages 18-64 with >=1 month parent/caretaker or childless adult eligibility between December 2018 – September 2020, and no eligibility due to pregnancy.	513,157
(2) Continuous enrollment from December 2018 – September 2020	115,638
(3) Diagnosis of OUD in any position on outpatient, inpatient, or emergency department claim, June 2018 – November 2018	6,435

© 2022 Tilhou AS, et al. *JAMA Health Forum.*

eAppendix 2

eTable 2. Comparison of Analytic Sample to Underlying Population of Adult Medicaid Beneficiaries with at least one month of Parent/Caretaker or Childless Adult eligibility, December 2018 – September 2020

	(1)<br>Population<br>**N= 513,157**	(2)<br>Continuously Enrolled Sample<br>**N=115,638**	(3)<br>Continuously Enrolled with OUD (Analytic Sample)<br>**N=6,435**
Average # months enrolled	14.43	22	22
Eligibility Category			
Childless Adult	266,911 (52%)	64,715 (56%)	3,974 (62%)
Parents/Caretakers	201,288 (48%)	50,923 (44%)	2,461 (38%
Age group			
18-34	49.86%	38.03%	43.92%
35-49	31.38%	37.08%	45.05%
>49	18.31%	24.89%	11.03%
Race			
American Indian	2.04%	2.04%	4.17%
Asian	3.08%	3.14%	0.40%
Black	20.12%	18.72%	7.10%
Hispanic	6.60%	5.95%	4.53%
Pacific Islander	0.10%	0.10%	0.02%
White	54.90%	62.90%	78.04%
Multiracial	5.16%	3.28%	3.69%
Geography			
Urban	69.23%	68.11%	69.57%
Rural	22.59%	23.67%	21.83%
Missing	8.18%	8.22%	8.59%
Education			
Less than High School	18.60%	18.35%	20.99%
High School or More	52.93%	54.92%	61.99%
Income			
<=50 FPL	65.82%	72.40%	85.53%
50-100% FPL	29.90%	27.60%	14.47%
>100% FPL	4.29%	0.00%	0.00%
Missing	0.01%	0.00%	0.00%

Notes:

Column (1) corresponds to the first row in eTable 1, Individuals ages 18-64 with >=1 month parent/caretaker or childless adult eligibility between December 2018 – September 2020, and no eligibility due to pregnancy. Column (2) corresponds to the second row in eTable 1, the subset of persons in the population with continuous enrollment from Dec 2018 – Sept 2020. Column (3) corresponds to the third row in eTable 1, the subset of the continuously enrolled sample with a diagnosis of OUD observed between June 2018-November 2018.ddd

© 2022 Tilhou AS, et al. *JAMA Health Forum.*

eAppendix 3. Comparison of Current Cohort to an Alternative Future Staggered Cohort

To assess the generalizability of our findings, we compared the study cohort to an alternative cohort identified three months forward (identified by claims 09/2018-02/2019 instead of 06/2018-11/2018). Here we show that sociodemographic characteristics are similar across the two cohorts.

eTable 3. Characteristics of the Current (identified 06/18-11/18) and Alternative (identified 09/18-02/19) Cohorts

	**Current Cohort**	**Alternative Cohort**
	N = 6,453<br>No. (%)	N = 7,006<br>No. (%)
Eligibility category		
Childless Adults	3986 (61.8)	4344 (62.0)
Parents	2467 (38.2)	2662 (38.0)
Age group		
18-34	43.89%	44.42%
35-49	45.08%	44.92%
>49	11.03%	10.66%
Race and Ethnicity		
American Indian	4.17%	4.30%
Asian	0.40%	0.39%
Black	7.10%	7.29%
Hispanic	4.53%	4.65%
Pacific Islander	0.02%	0.03%
White	78.04%	77.58%
Multiracial	3.69%	3.80%
Sex		
Female	53.23%	53.07%
Geography		
Urban	70.26%	70.17%
Rural	21.20%	21.42%
Education		
Less than High School	20.99%	19.91%
High School or more	61.99%	61.99%
Income		
<=50 FPL	85.54%	85.43%
50-100% FPL	14.46%	14.57%
>100% FPL	0.00%	0.00%

© 2022 Tilhou AS, et al. *JAMA Health Forum.*

Non-constant demographic variables are counted from first observed month in sample period. Abbreviations: OTP, opioid treatment program; UDT, urine drug test; FPL, federal poverty level.<br>Missing, current cohort: race, 133 (2.1%), geography, 551 (8.5%), and education, 1156 (17.9%)<br>Missing, alternative cohort: race, 137 (1.96%), geography, 589 (8.41%), and education, 1268 (18.10%)	

© 2022 Tilhou AS, et al. *JAMA Health Forum.*

eAppendix 4. Definition of Outcome Measures

Opioid Treatment Program Engagement
We defined OTP engagement for each person week. This binary variable takes on a value of 1 if the individual had any claim during the week in which the provider taxonomy code for the billing provider was, 261QM2800X - Clinic/Center Methadone Clinic. Absent a claim with this type of billing provider, the OTP variable takes a value of 0.

Office Based Opioid Treatment
We defined this binary variable for each person-week. We defined treatment as a prescription medication claim for buprenorphine or buprenorphine/naloxone. We determined the days supplied using the start and end dates of each prescription, and aligned those days supplied to each week. The OBOT variable takes on a value of 1 if the days supplied is >=1 in the person-week and 0 otherwise. We followed the methods published by Medicaid Outcomes Distributed Research Network (MODRN) to address overlap in medications.[1]

Urine Drug Test
We defined this binary variable for each person-week. We adopted the code set used by the MODRN to identify urine drug tests (shown below).[1]

"80100" "80101" "80102" "80103" "80104" "80299" "80300" "80301" "80302" "80303" "80304" "80305" "80306" "80307" "80320" "80321" "80322" "80323" "80324" "80325" "80326" "80327" "80328" "80329" "80330" "80331" "80332" "80333" "80334" "80335" "80336" "80337" "80338" "80339" "80340" "80341" "80342" "80343" "80344" "80345" "80346" "80347" "80348" "80349" "80350" "80351" "80352" "80353" "80354" "80355" "80356" "80357" "80358" "80359" "80360" "80361" "80362" "80363" "80364" "80365" "80366" "80367" "80368" "80369" "80370" "80371" "80372" "80373" "80374" "80375" "80376" "80377" "82660" "83925" "83992" "84311" "G0430" "G0431" "G0434" "G0477" "G0478" "G0479" "G0480" "G0481" "G0482" "G0483" "G0659" "G6045" "G6046" "G6053" "G6056" "G6058" "H0003" "H0048" "H0049" "Z2103" "Z2104" "Z2105" "Z2106"

We assigned a value of 1 to the UDT outcome variable if the UDT occurred in an outpatient setting and zero otherwise to increase the likelihood of capturing only UDTs associated with OBOT.

We used the following method to identify the UDT setting. We defined the setting to be an OTP if the subject had an outpatient visit where the billing provider was an OTP on the same day as the UDT. We defined the source to be an ED if the subject had an ED visit on the same day as the UDT. We defined the source to be inpatient if the subject was hospitalized on the date of the UDT. We defined the source to be outpatient if the subject had an outpatient visit for which the billing provider was *not* an OTP. We defined the source as unknown (and presumed outpatient) if on the date of the UDT, the subject did not have an OTP, ED, inpatient or outpatient encounter. If the person had a health care encounter at more than one setting on the date of the UDT, we assigned the setting in this order: OTP, inpatient, ED, outpatient.

© 2022 Tilhou AS, et al. *JAMA Health Forum.*

eAppendix 5. Definition of Person-Week

We divided each month into four quarters with the aim of having the same number of days per quarter within the month to the extent possible. We assigned days that could not be evenly divided across quarters within the month as shown below.

eTable 4. Days per month-quarter for each calendar month

Months	Total Days	# Days, Q1	#Days, Q2	#Days, Q3	#Days, Q4
Jan, March, May, July, August, October, December	31	8	7	8	8
April, June, September, November	30	8	7	7	8
February 2019	28	7	7	7	7
February 2020	29	7	7	7	8

Since month-quarters can vary between 7 and 8 days, we controlled for this variation in our models. Results did not change for any of the outcomes. Thus, our final models are unscaled.

© 2022 Tilhou AS, et al. *JAMA Health Forum.*

REFERENCES

1. The Medicaid Outcomes Distributed Research Network (MODRN). Use of Medications for Treatment of Opioid Use Disorder Among US Medicaid Enrollees in 11 States, 2014-2018. *JAMA*. 2021;326(2):154-164. doi:10.1001/jama.2021.7374.

© 2022 Tilhou AS, et al. *JAMA Health Forum.*
